# Extracellular vesicles released from blood-brain barrier endothelial cells mediate brain iron accumulation during LPS-induced brain inflammation

**DOI:** 10.1016/j.jbc.2026.111399

**Published:** 2026-03-21

**Authors:** Kondaiah Palsa, Timothy B. Helmuth, Aurosman Pappus Sahu, Yuanjun Zhao, Rebecka O. Serpa, Elizabeth B. Neely, Irina A. Elcheva, Vladimir S. Spiegelman, James R. Connor

**Affiliations:** 1 Department of Neurosurgery, Penn State College of Medicine, Hershey, Pennsylvania, USA; 2 Department of Pediatrics, Penn State College of Medicine, Hershey, Pennsylvania, USA; 3 Cell and Biological Systems Department, Penn State College of Medicine, Hershey, Pennsylvania, USA

**Keywords:** blood-brain barrier endothelial cells, extracellular vesicles, H-ferritin, inflammation, iron

## Abstract

Brain inflammation leads to an increase in the amount of iron in brain tissue, but the source of the iron that could lead to accumulation remains unclear. Our recent *in vitro* study discovered that, in addition to the traditional pathway, iron can be released from the blood-brain barrier *via* extracellular vesicles (EVs). Herein, we investigated the impact of brain inflammation on iron release *via* EVs from the brain microvasculature (BMV). For this study, we induced brain inflammation in three-month-old C57BL/6 by intracerebroventricular injection of lipopolysaccharide (LPS, 12 μg/mice). For *in vitro*, we used human blood-brain barrier endothelial cells derived from human-induced pluripotent stem cells (hiPSCs). The LPS injection activated microglia and astrocytes as well as increased brain proinflammatory cytokines compared to the control mice. Furthermore, brain inflammation increased the iron levels in the brain parenchyma but decreased the iron levels in BMV. Brain inflammation was associated with the degradation of ferroportin (FPN1), an iron exporter, in the BMV. CD63, an EVs membrane protein, was increased in the BMV and associated with increased FTH1 release *via* EVs from BMVs to the brain. Moreover, brain inflammation decreased iron in BMV as evidenced by an increase in the transferrin receptor and decreased FTH1, suggestive of increased iron uptake. Pharmacological reduction of EVs by GW4869 reduced iron accumulation in the inflamed brain parenchyma compared to control mice. In summary, we have discovered a novel mechanism by which BMV-released EVs enriched with FTH1 and reveal a significant mechanism for brain iron accumulation during inflammation.

Iron is crucial for various biological functions, particularly in cognition and brain health. It acts as an electron donor, acceptor, and oxygen carrier, making it essential for oxidation-reduction reactions critical to cellular metabolism (1). While iron benefits the brain, fluctuations in its levels can lead to neurological problems (2). Blood-brain barrier (BBB) endothelial cells play a key role in iron transport to the brain and thus regulate its availability to the brain (3, 4). The transferrin receptor1(TfR1) on the apical side of endothelial cells in the blood–brain barrier takes up iron bound to transferrin and releases it to the brain parenchyma as free iron *via* the iron exporter ferroportin1/hephaestin (FPN1/HEPH) or iron-protein complex (3, 5). The FPN1 released iron is regulated by hepcidin, which is released from astrocytes and microglia (6, 7, 8). The release of EVs is iron-regulated by the iron concentration in the endothelial cells of the BBB (9). However, during inflammation, this process is altered in a way that is not known and addressed in this study.

Inflammation is the primary mechanism for iron accumulation and the progression of neurological disorders (10). Glial cells, especially microglia, play the pivotal role in inflammation (11). Under physiological conditions, microglia mainly eliminate metabolic products and toxic materials. Activated microglia, however, increase the release of proinflammatory cytokines, which leads to neuronal death (12). To induce brain inflammation, studies activate the microglia by using Lipopolysaccharide (LPS), a component that stimulates the immune response *via* the Toll-like receptor four (TLR-4) (13). TLR-4 is primarily found on microglia in the central nervous system (14), and activation of the microglia produces proinflammatory cytokines such as TNF-α, IL-6, and IL-1β (15). These cytokines serve as critical mediators of neuroinflammation. LPS administration is frequently employed to investigate diseases associated with neuroinflammation in mouse models (16). However, the specifics of iron trafficking within blood-brain barrier (BBB) endothelial cells during brain inflammation remain unclear, particularly whether inflammation affects iron transport across the intact BBB.

Our current study aims to investigate the effect of brain inflammation on iron uptake in blood-brain barrier endothelial cells. To this end, we induced brain inflammation through intracerebroventricular (ICV) injection of LPS and examined the iron homeostasis at the brain microvasculature. This study addresses a gap in knowledge regarding the mechanism by which inflammation alters iron flux across the intact BBB.

## Results

### ICV injection of LPS activates the microglia and astrocytes

**Figure 1:**
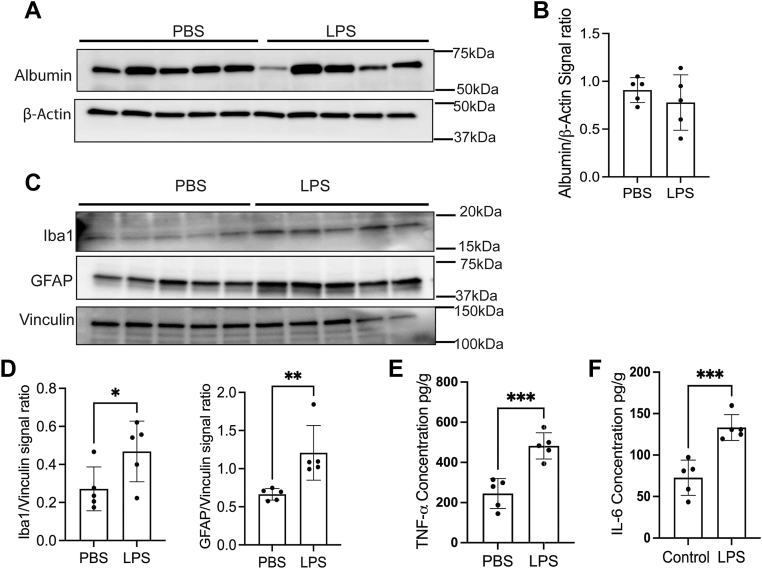
**ICV injection of LPS activates the microglia and astrocytes.** Twelve-week-old male mice were ICV injected with PBS or LPS and incubated for 48 h. *A* and *B*, the immunoblot illustrates parenchyma Albumin levels in PBS and LPS-injected groups, and β-Actin is present as the loading control. *C* and *D*, illustrates immunoblot of the GFAP and Iba1, and Vinculin is present as the loading control. Minimum n = 5 used for this experiment. *E* and *F*), bar graphs demonstrate the concentration of TNF-α and IL-6 levels in the parenchyma of PBS and LPS-injected groups. n = 6 for all conditions, the data were presented as mean ± SD, and a student unpaired *t* test was used for the statistical significance. ∗*p* < 0.05, ∗∗*p* < 0.01, ∗∗∗*p* < 0.001.

To induce brain inflammation, we injected 12 μg of LPS into the brain *via* ICV (16). After 48 h, we perfused the mice with 0.1 M PBS, and the brains were collected. The brain was homogenized and separated into BMV and parenchymal fractions. First, we determined the permeability of the BBB by injecting the Evans blue dye and measuring the albumin levels in the brain. Our results demonstrated that Evans blue dye was not detected in the brains following ICV injection of either LPS or PBS (Fig. S1). Next, we measured the albumin levels in the brain parenchyma and found no significant changes (Fig. 1, *A* and *B*). The glial fibrillary acidic protein (GFAP) and ionized calcium-binding adaptor molecule 1 (Iba1) are the markers for astrocytes and microglia, respectively, whose expression is increased when these cells are activated (16, 17). Therefore, we measured Iba1 and GFAP expression and found that LPS-injected mice have increased expression of GFAP and Iba1 compared to the PBS group (Fig. 1, *C* and *D*). Additionally, we measured the proinflammatory cytokine levels in the parenchyma by ELISA. The TNF alpha and IL-6 levels were significantly increased in LPS-injected mice compared to the PBS mice (Fig. 1, *E* and *F*).

### LPS-induced brain inflammation alters BMV iron, transferrin receptor, and ferroportin

**Figure 2:**
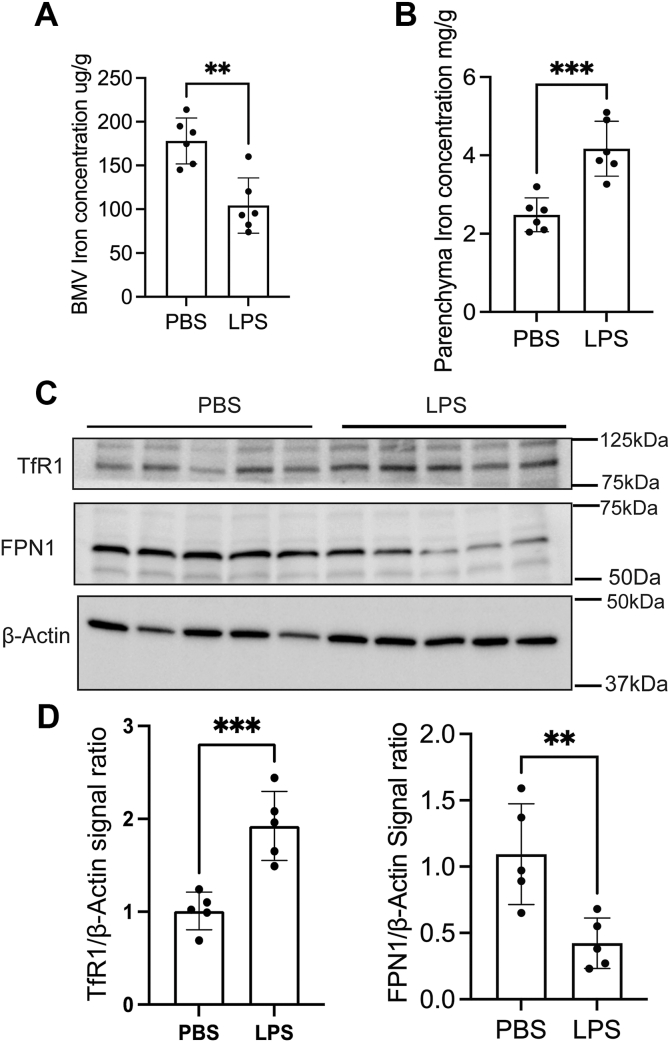
**LPS-induced brain inflammation alters the BMV irontransferrin receptor, and ferroportin.** Twelve-week-old mice were ICV injected with PBS or LPS and incubated for 48 h. After that, we collected the brain and separated in to the brain parenchyma and BMV. *A*, the bar graph represents the Iron concentration in the BMV. *B*, illustrates the iron concentration in the parenchyma. *C* and *D*, immunoblot and their bar graphs represent the BMV TfR1 and FPN1 expression, β-Actin is present as the loading control. Minimum n = 5 used for these experiment conditions, the data were presented as mean ± SD, and a student unpaired *t* test was used for the statistical significance. ∗∗*p* < 0.01, ∗∗∗*p* < 0.001.

To determine whether brain inflammation affects iron levels in the BMV and parenchyma, we assessed the iron concentration in these areas using ICP-MS. LPS-induced brain inflammation significantly reduced iron levels in the BMV but increased levels in the parenchyma (Fig. 2, *A* and *B*). The iron concentrations in the BMV and parenchyma are dependent on the TfR1 expression. We observed a notable increase in TfR1 expression in the BMV in the LPS group compared to the PBS group (Fig. 2, *C* and *D*). Additionally, we measured the free iron exporter FPN1 expression, which is significantly lower in the BMV of the LPS group than in the PBS-injected mice (Fig. 2, *C* and *D*). In summary, LPS-induced brain inflammation results in decreased iron levels in the BMV but not in the brain parenchyma.

### CD63 expression is increased in BMV during LPS-induced brain inflammation

**Figure 3:**
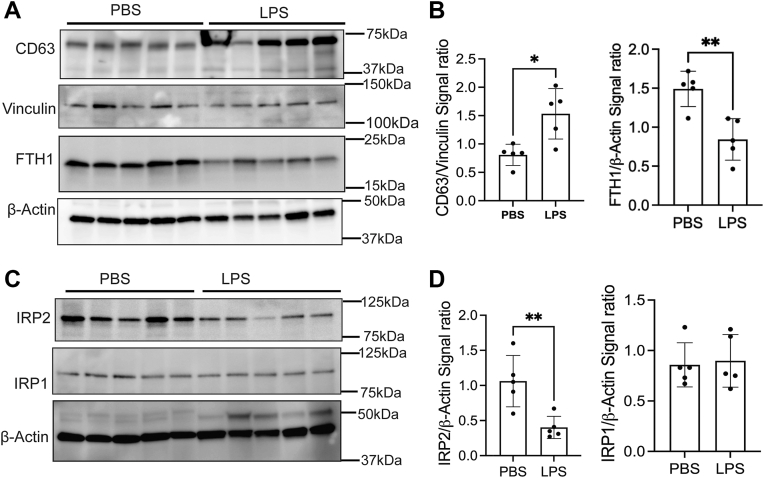
**CD63 expression increased in BMV during LPS-induced brain inflammation.** Twelve-week-old mice were ICV injected with PBS or LPS and incubated for 48 h. After that, the collected brain is separated into the brain parenchyma and BMV. *A* and *B*, immunoblot and bar graphs represent the CD63 and FTH1 expression in BMV, and vinculin and β-Actin are present as the loading control, respectively. Minimum n = 5 used for these experiments. *C* and *D*, immunoblot and bar graphs illustrate the IRP1 and IRP2 expressions in BMV; β-Actin is present as the loading control. Minimum n = 5 used for these experiment conditions, the data were presented as mean ± SD, and a student unpaired *t* test was used for the statistical significance. ∗*p* < 0.05 ∗∗*p* < 0.01.

Our prior research demonstrated that CD63^+^ EVs released by ECs mediate FTH1 and iron transport to the brain, and the EV formation is regulated by the iron concentration in ECs (9). We observed a significant increase in CD63 expression compared to the control group following the LPS injection in the BMV (Fig. 3, *A* and *B*). Additionally, we measured the FTH1 levels in the BMV of PBS and LPS-injected groups. Brain inflammation was associated with significantly decreased expression of FTH1 in BMV compared to the PBS group (Fig. 3, *A* and *B*). The FTH1 and CD63 expression are regulated by the IRPs in the ECs (9, 18). Thus, we assessed Iron regulatory protein (IRP) expression; brain inflammation reduced IRP2 expression in BMV, but it did not impact IRP1 expression (Fig. 3, *C* and *D*). These findings indicate that LPS-induced brain inflammation disrupts iron regulatory mechanisms in the BMV; despite lower iron levels, IRP2 expression remained low.

### LPS-induced brain inflammation increases the release of CD63+EVs from the ECs of the BBB

**Figure 4:**
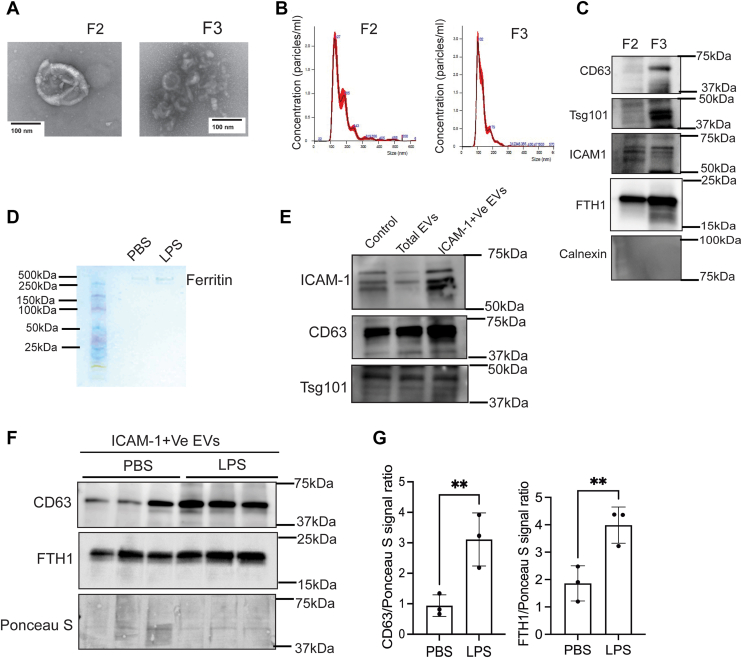
**LPS-induced brain inflammation increases the release of CD63+EVs from the ECs of the BBB.** Twelve-week-old mice were ICV injected with PBS or LPS and incubated for 48 h. After that, we isolated EVs from the whole brain by density gradient centrifugation. *A*, the TEM image illustrates the size of the EVs in F2 and F3 fractions. *B*, nanoparticle tracking analysis of F2 and F3 fractions. *C*, immunoblot illustrates the EVs markers expression in F2 and F3 fractions- CD63, Tsg101, FTH1, ICAM-1, and calnexin. *D*, *Prussian blue* staining of PBS and LPS EVs associated ferritin iron, each band represents the pooled sample of the n = 3. (*E*). Immunoblot represents the ICAM-1+ EVs isolation from the whole EVs. Here, control means the EVs were incubated with IgG2b, κ, and whole EVs means ICAM-1 depleted EVs. *F* and *G*, immunoblot represents the FTH1 and CD63 expression in the ICAM-1+ve EVs and Ponceau S, as used as a loading control. n = 6 for all conditions, each band represents the pooled sample of the n = 2, data were presented as mean ± SD, and a student unpaired *t* test was used for the statistical significance. ∗∗*p* < 0.01.

To determine if brain inflammation is associated with increased release of CD63+EVs from endothelial cells (ECs) and FTH1, we isolated EVs from the whole brain and then assessed the expression of CD63 and FTH1 in the EVs released by ECs. Initially, the EVs were isolated from the normal brain by using density gradient centrifugation. Subsequently, we verified that the isolated particles were indeed EVs by evaluating their size and shape through transmission electron microscopy (TEM), nanoparticle tracking analysis (NTA), as well as checking specific markers *via* immunoblotting across three fractions. We found that fractions F2 and F3 contained EVs, with F2 having larger EVs, lower concentration, and F3 containing smaller and higher EVs concentration (Fig. 4, *A* and *B*). The EVs are two types based on their sizes, which are small EVs or exosomes (30–150 nm), and macrovesicles (200nm - 1μm) (19). In present study, the isolated vesicles size is 30 to 150 nm in size. We then analyzed the expression of EV markers and associated proteins—CD63, Tsg101, intercellular adhesion molecule 1 (ICAM-1), and FTH1 within the F2 and F3 fractions (Fig. 4*C*). Notably, the levels of EV markers and FTH1 were higher in the F3 fraction, prompting us to utilize this fraction for further studies. Next, we performed Prussian Blue staining to see whether the EVs associated with FTH1 contain iron. The brain inflammation increased the release of iron-rich FTH1 in EVs compared to the control group (Fig. 4*D*). To identify whether the ECs of BBB increase the release of FTH1 in EVs, we focused on ICAM-1, a marker exclusively expressed in on EVs of endothelial cells and increases during inflammation (20). We isolated ICAM-1+EVs from the total brain EV population (Fig. 4*E*). Here, the control is an IgG2b, κ, antibody added to EVs. Next, we measured the expression of FTH1 and CD63 in ICAM-1+EVs. Results showed that the expressions of CD63 and FTH1 were significantly higher in the group of mice injected with LPS compared to those injected with PBS (Fig. 4, *F* and *G*).

### The proinflammatory cytokines increase the iron release from the BBBECs *via* EVs *in vitro*

**Figure 5:**
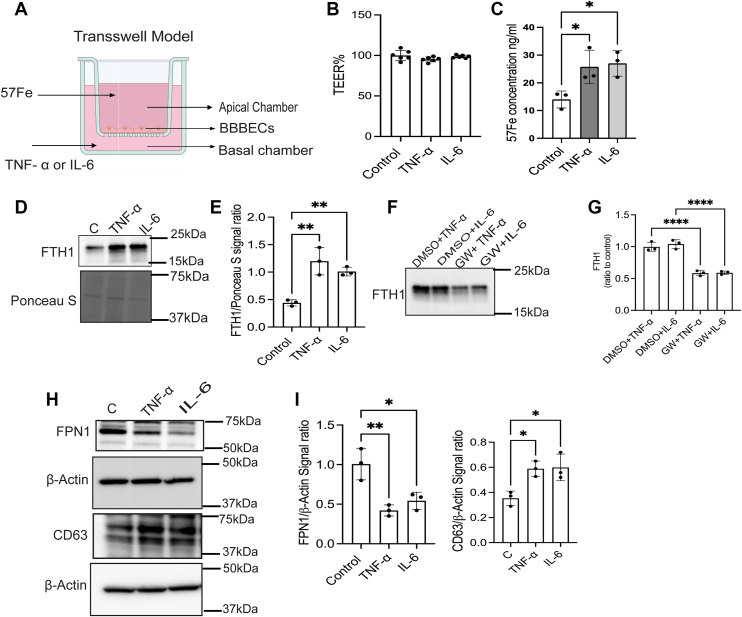
**The proinflammatory cytokines increase the iron release from the BBBECs *via* EVs.** ECs were grown on 0.4 μm pore size filters. ^57^Fe-ascorbic acid (100 μM) was added to the apical chamber and incubated for 24 h. After 24 h ^57^Fe was removed from the apical chamber, and TNF-α or IL-6 was added to the basal chamber and incubated for 24 h. EVs were isolated from the basal chamber media, and ^57^Fe activity was measured in EVs. For the EVs inhibition experiment, GW4869 and DMSO (control) were added to the apical side and pre-incubated for 6 h, after that TNF-α and IL-6 were added to the basal side and incubated for 24 h. EVs were isolated from the basal media following incubation. *A*, illustrates the transwell experimental model. *B*, illustrates the permeability of BBBECs by measuring TEER values in control and proinflammatory cytokine-treated cells. *C*, the bar graph illustrates the ^57^Fe concentration in control vs proinflammatory cytokines-treated cells. The experiment was performed in three independent experiments; three technical replicates were performed in each independent experiment. The data are shown as the mean ± S.D. *D* and *E*, the immunoblot and bar graph demonstrate the EVs FTH1 levels, and Ponceau S, used as a loading control. The experiment was performed in three independent experiments; three technical replicates were performed in each independent experiment. The data are shown as the mean ± S.D. *F* and *G*, immunoblot and bar graph representing the EVs FTH1 expression in DMSO (control) and GW4869 under proinflammatory cytokines treatment on the basal side. FTH1 expression was normalized to the control, where the control is DMSO-treated cells. The experiment was performed in three independent experiments; three technical replicates were performed in each independent experiment. The data are shown as the mean ± S.D. *H* and *I*, immunoblot and bar graphs demonstrate the FPN1 and CD63 expression in ECs in the presence or absence of proinflammatory cytokines, β-Actin is present as the loading control. The experiment was performed in three independent experiments; three technical replicates were performed in each independent experiment. The data are shown as the mean ± S.D., and one-way ANOVA followed by Tukey's *post hoc* analysis was used for the statistical significance ∗*p* < 0.05. ∗∗*p* < 0.01, ∗∗∗∗*p* < 0.0001.

To further confirm ECs of BMV increase the iron release by EVs during brain inflammation, we treated the ECs with proinflammatory cytokines. For this experiment, ECs were loaded with ^57^Fe to the apical side and treated with TNF-α (10 ng/ml) and IL-6 (50 ng/ml) in the basal chamber (Fig. 5*A*). ^57^Fe was added to the apical chamber in the absence of proinflammatory cytokines in the basal chamber as a control. After 24 h, we measured the permeability of ECs by TEER values; the proinflammatory cytokines did not affect the permeability of the ECs at the 24 h time point (Fig. 5*B*). After measuring TEER values, EVs were isolated from the basal chamber media, and ^57^Fe and FTH1 activity were measured in EVs. The proinflammatory cytokines significantly increased the ^57^Fe concentration and FTH1 compared to the control in EVs (Fig. 5, *C*–*E*). To further confirm that EV-associated FTH1 mediates iron transport to the brain side, EV release from endothelial cells was inhibited with GW4869, followed by the treatment of proinflammatory cytokines on the basal side for 24 h. EVs were isolated from the basal medium, and FTH1 levels were measured; EV inhibition significantly reduced FTH1 levels compared with controls (Fig. 5, *F* and *G*). Next, we assessed FPN1 and CD63 expressions in endothelial cells under inflammatory and control conditions to determine whether inflammatory cytokines affect endothelial iron exporters. The exposure to proinflammatory cytokines significantly decreased the FPN1 expression and increased the CD63 levels compared to the control (Fig. 5, *H* and *I*).

### EVs inhibition by GW4869 reduces the LPS-induced brain iron accumulation

**Figure 6:**
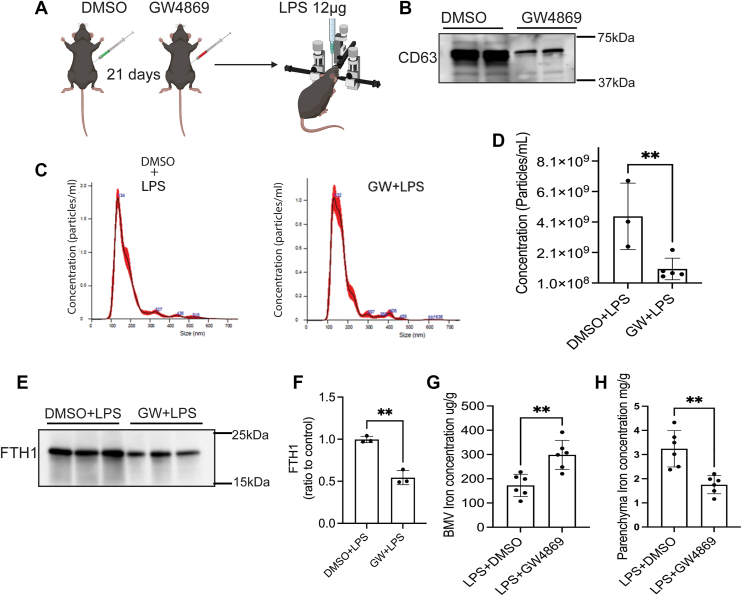
**EVs inhibition by GW4869 reduces the LPS-induced brain iron accumulation.** Eight-week-old mice were intraperitoneally injected with either DMSO or GW4869 for 3 weeks to inhibit the EVs synthesis in ECs of BMV. After 3 weeks, mice were ICV injected with LPS and incubated for 48 h; DMSO or GW4869 were continued during and after LPS injections. After that brain was separated into BMV and parenchyma, and the iron concentration was measured by ICP-MS. ICAM-1–positive extracellular vesicles were isolated from whole-brain EVs derived from control (DMSO) and GW4869-injected inflamed brains. Minimum n = 3 used for the experiment. *A*, illustrates experimental design. *B*, immunoblot demonstrates the CD63 expression in whole brain EVs, n = 2 mice. *C*, represent the images of NTA of ICAM1- + Ve EVs, and (*D*). Bar graph represents the concentrations of ICAM-1+ve EVs in control (DMSO + LPS) and GW4869. E and F, immunoblot illustrates the FTH1 expression in ICAM-1+Ve EVs in control and GW4869 injected groups in the inflamed brain. FTH1 expression was normalized to the control, where the control is DMSO + LPS. *G*, bar graph illustrates the BMV iron concentration. *H*, bar graph demonstrates the parenchyma iron concentration. n = 6 for all conditions, the data were presented as mean ± SD, and a student unpaired *t* test was used for the statistical significance. ∗∗*p* < 0.01.

We next inhibited the synthesis of EVs to determine if LPS-induced parenchyma iron accumulation was mediated by EVs. To achieve this, we administered GW4869 intraperitoneally for 3 weeks (21 days) and then injected LPS *via* ICV (Fig. 6*A*). After 48 h post-LPS injection, we collected and separated the brains into BMV and parenchyma, and measured the iron concentrations. Before LPS injection, we tested whether GW4869 inhibited EVs release into the brain. After 21 days of IP injections, we measured the CD63 expression in whole brain EVs. 21 days of IP injection of GW4869 significantly decreased the whole brain EVs, as confirmed by the CD63 expression in total EVs (Fig. 6*B*). To determine whether GW4869 reduces endothelial cell–derived EVs from BMV during inflammation, ICAM-1–positive EVs were isolated from total brain EVs and quantified by NTA. GW4869 treatment significantly decreased EV concentration compared with controls of the inflamed brain (Fig. 6, *C* and *D*). The above results showed that endothelial cell–derived EVs and their associated FTH1 contribute to brain iron accumulation during inflammation, so we next measured FTH1 levels in ICAM-1–positive EVs, which were also significantly lower in the GW4869 group than in controls (Fig. 6, *E* and *F*). Next, we measured the iron concentration in BMV and the parenchyma of EVs inhibited in an inflamed brain. EVs inhibition resulted in increased iron in the BMV group during LPS-induced inflammation compared to those in the LPS alone group (Fig. 6*G*). In contrast, the iron level in the parenchyma was significantly decreased when EVs were inhibited during inflammation of the EVs inhibition compared to the LPS group alone (Fig. 6*H*).

## Discussion

Brain Iron accumulation is a common symptom of neurodegenerative disorders, such as Parkinson's disease (PD), amyotrophic lateral sclerosis, and Alzheimer's disease (AD) (21, 22, 23, 24). The potential mechanism regulating this brain iron accumulation is inflammation (25). In the brain during inflammation, proinflammatory cytokines are elevated, leading to a decrease in FPN1, an iron exporter in EC, and a potential reduction in iron release (26, 27, 28, 29, 30). However, despite this down-regulation of the major iron exporter in the BMV, iron accumulates in the brain. Previous studies showed that iron is also transported from ECs as free and protein-bound iron (4, 9). In this study, we investigated the effects of brain inflammation on BMV iron levels and the expression of their iron transporters. We demonstrated that FTH1 is associated with EVs released from ECs and contains iron. When released during LPS-induced brain inflammation, it could lead to brain iron accumulation. The EV iron release is independent of the hepcidin/FPN1 system. For this study we first confirmed that ICV injection of LPS induces inflammation in the brain. Previous studies showed that microglia in the brain express the LPS receptor; the injected LPS binds to this receptor and activates the microglia (31, 32). The activated microglia release proinflammatory cytokines, which further activate the astrocytes (33). The current results, consistent with previous studies, demonstrate that ICV injection of LPS activated microglia and astrocytes, increasing the release of proinflammatory cytokines (IL-6 and TNF-α). The activated astrocytes and released proinflammatory cytokines affect the iron homeostasis and regulation in the blood-brain barrier ECs. In the present study, ICV LPS-induced inflammation did not alter BBB permeability for larger molecules, as evidenced by similar albumin levels in both the LPS and PBS injected mouse groups. These results are concordant with a previous study, ICV injection of LPS did not increase the permeability for larger molecules (34).

As previously mentioned, despite the crucial role of endothelial cells in iron transport to the brain, whether inflammation increases or decreases iron flux across the BBB remains unknown. In the present study, we found that FPN1 expression levels in the BMV of LPS-injected mice and BBBECs of proinflammatory cytokines-treated were decreased compared to those in the PBS group, which is aligned with previous studies that LPS induces brain inflammation by releasing proinflammatory cytokines, which degrade FPN1 and elevate TfR1 (26, 35). However, current study shows that iron levels were significantly decreased in BMV, which, based on previous studies and ours, would be expected to accumulate due to the iron export protein is degraded. Additionally, the increase in TfR1 expression and decreased FTH1 in the BMV compared to controls suggests that iron uptake is increased and exported through FTH1 as an alternative pathway to FPN to the brain during inflammation. We recently reported that endothelial cells release iron to the brain *via* EV by regulating CD63 (9). Herein, CD63 expression levels in the BMV were found to be higher in the inflamed brain compared to controls; CD63 serves as a marker for EVs, critical for their synthesis and release. CD63 has a 5’ iron-responsive element (IRE) sequence regulated by iron regulatory proteins (IRPs) (18). The expression of these IRPs depends on cellular iron concentration and is inversely related to iron levels in the cells (36). Conversely, our study demonstrated a decrease in IRP2 expression, even though the BMV exhibited lower iron levels, FTH1, which indicates that brain inflammation dysregulates the iron homeostasis in ECs of BMV, inducing CD63 protein expression and, subsequently, EV formation and release. Prior studies indicate that during inflammation, iron metabolism becomes dysregulated, with increased TfR1 expression and reduced IRP2 expression (37, 38). The possible explanation for this IRP2 degradation during LPS injection may be that LPS induces iNOS expression, which increases nitric oxide production. Elevated nitric oxide S-nitrosylates the IRP2, which promotes its ubiquitination and degradation by the 26S proteasome (39). Our previous work showed that FTH1 is involved in the iron transport to the brain during brain development and ID conditions (40, 41, 42, 43). Also, FTH1 crosses the blood-brain barrier and is transported intact to the brain cells (44). During inflammation, the release of ferritin into the circulation increases (45). Furthermore, studies demonstrated that ferritin (H and L) is released in EVs, which are loaded with iron (18, 46). Our *in vitro* study showed that ECs release the FTH-iron *via* EVs to the brain (9). Zhang *et al*. demonstrated that inflammation increases the release of the iron-rich FTH1 *via* EVs from the intestinal epithelial cells to the blood. The released EVs' ferritin iron increases the inflammatory response in macrophages (47). In the current study, consistent with a previous study, the LPS-induced brain inflammation increased the release of EVs FTH1-iron levels from ECs of BMV in both *in vitro* and *in vivo* studies. These EV-associated FTH1 levels corresponded to parenchyma iron levels during LPS-induced brain inflammation. During inflammation, ECs increase the uptake of iron and release FTH1-iron through the EVs to the brain. Additionally, EVs inhibition by GW4869 decreases the iron accumulation in the parenchyma and increases the iron levels in BMV. These EV inhibition results further confirm that ECs of BMV release EVs, mediating the iron accumulation in the brain during inflammation.

Based on our findings, we conclude that LPS-induced brain inflammation disrupts iron homeostasis within the BMV. This disruption in iron homeostasis in the BMV results in overproduction and release of ferritin-enriched EVs from the ECs, resulting in iron accumulation in the brain parenchyma. This is the first demonstration that ECs of the BMV release EVs that are involved in iron accumulation in the brain during inflammation. The mechanism by which the iron accumulation *via* EVs is occurring may be *via* the enrichment of FTH1 in the EVs, and offers an explanation for how brain iron accumulation can occur during inflammation, bypassing the hepcidin/FPN1 regulatory mechanism. This novel EV-mediated FTH1 transport pathway could provide opportunities to explore mechanisms for the delivery of therapeutic compounds to the brain during the disease process, which typically involves inflammation. Also, these findings suggest potential targets for future clinical applications in patients with iron-accumulated neurodegenerative disease.

## Experimental procedures

### Animals and treatments

Male C57BL/6J mice, aged 11 to 12 weeks, were obtained from the Jackson Laboratory. All animal experiments received approval and were conducted following the guidelines of Penn State University's Animal Ethics Committee (#PROTO201900998). The mice were housed in an environment with automatically regulated temperature (21–25 °C), relative humidity (45–65%), and a light-dark cycle of 12 h. We divided mice into two groups: 1) i.c.v. saline group, or 2) i.c.v. LPS (12 μg) group, with each group comprising six male mice (n = 6). The i.c.v. injections of 12 μg LPS (diluted in 3 μl saline) and saline (as a control) were performed on the same day using a microsyringe, following stereotaxic coordinates −2.6 mm dorsal/ventral, −1.5 mm lateral, and −0.2 mm anterior/posterior from bregma as described previously (16).

### EVs inhibition model

GW4869 (N, N’-Bis[4-(4,5-dihydro-1H-imidazol-2-yl) phenyl]-3,3’-p-phenylene-bis-acrylamide dihydrochloride) is the most widely used pharmacological agent to block EVs generation and reduce EVs release by neutral sphingomyelinase (nSMase-2). The nSMase-2 activity is important for creating the large lipid raft domains involved in EVs shedding, as a result, inhibition of nSMase-2 reduces the release of EVs from the cells. GW4869 was prepared and injected as described previously (48, 49, 50). GW4869 was dissolved in DMSO at 5 mg/ml. Mice were injected intraperitoneally with 200 μl of 0.3 mg/ml GW4869 in 0.9% normal saline (60 μg/mouse; 2–2.5 μg/g body weight) or 200 μl of 3.75% DMSO as a saline control every 24 h for four weeks (28 injections total). For EV inhibition in ECs, GW4869 (20 μM) was applied to the apical side for 6 h prior to basolateral treatment with proinflammatory cytokines. Following this pre-treatment, proinflammatory cytokines were added to the basolateral compartment, and ECs were incubated with the inhibitor for an additional 24 h.

We used the same set of animals for PBS and LPS ICV injection study to measure the BMV, parenchyma iron concentration, and immunoblotting, n = 6 per group (half of the volume used for each). For EV isolations, we used *n* = 10 per group, n = 6 used for immunoblotting, two used for the EVs validation, and two used for the ferritin iron staining. For the LPS with EVs inhibition group, we used n = 10 for each group. n = 6 for the LPS injection and four were used for the validation of EVs inhibition.

### BBBECs derived from human induced pluripotent stem cells

Human endothelial cells were differentiated from ATCC-DYS0100 hiPSCs (human induced pluripotent stem cells) as described previously (9, 51). hiPSCs were seeded onto a Matrigel-coated plate in E8 medium (Thermo Fisher Scientific, 05990) containing 10 μM ROCK inhibitor (Y-27632, R&D Systems; 1254) at a density of 18,000 cells/cm2. The iPSCs differentiation was initiated by changing the E8 medium to E6 medium (Thermo Fisher Scientific, A1516401) after 24 h of seeding. E6 medium was changed every 24 h, and cells were continued to E6 medium for up to four days. After four days, cells were switched to basal endothelial medium (hESFM) (Thermo Fisher Scientific, 11111) supplemented with 10 nM bFGF (Fibroblast growth factor, Peprotech # 100–18B) and 10 μM RA (retinoic acid, Sigma R2625) and 1% B27 (Thermo Fisher Scientific, 17504–044). The medium was not changed for 48 h. After 48 h, cells were collected and replated onto transwell filters or cell culture plates coated with collagen IV and fibronectin. Twenty-four hours after replating, bFGF and RA were removed from the medium to induce the barrier phenotype.

### Isolation of brain microvasculature

The capillary depletion method was conducted as previously described (52). Briefly, fresh brain pieces were homogenized on ice by ten strokes with a Dounce homogenizer (smaller diameter pestle) in 3.5 ml HBSS and then centrifuged for 10 min at 1000*g*. The pellet was resuspended in 2 ml of 17% dextran (mol wt 60,000; 31397; Sigma-Aldrich) and centrifuged for 15 min at 4122*g* to separate the parenchymal cells from the vasculature. The top myelin and parenchymal cell layers were removed together and diluted with HBSS, then centrifuged for 15 min at 4122*g* to pellet the parenchymal cells. The vascular pellet was passed through a 100-μm mesh filter to purify the vasculature. Both vascular pellet and parenchymal cell pellets were resuspended in cold 1% NP40 in PBS with protease and phosphatase inhibitors, sonicated for 30 s, and then incubated for 20 min on ice. The cell lysate was collected after centrifugation for 10 min at 12,700*g*. The total protein concentration of each sample was measured using a BCA assay.

### EVs enrichment from tissue

Brain EVs isolation was performed as described previously (53). Modified briefly, brains were sliced on ice using a razor blade to generate 1 to 2 cm long, 2 to 3 mm wide sections of brain. The sliced brains were transferred to a 50 ml tube containing 75 U/ml of collagenase type III in Hibernate-E (at a ratio of 800 μl per 100 mg of brain). The tissues were incubated in a shaking water bath at 37 °C for a total of 20 min. The dissociated tissue was spun at 300*g* for 5 min at 4 °C. The supernatant from the 300*g* spin was then transferred to a fresh tube and spun at 2000*g* for 10 min at 4 °C. Finally, the supernatant was spun at 10,000*g* for 30 min at 4 °C. The supernatant was overlaid on a triple sucrose cushion (0.6 M, 1.3 M, 2.5 M). To make the triple sucrose cushion, 0.5 ml of 2.5 M sucrose (F4) was overlaid with 0.6 ml of 1.3 M sucrose (F3) followed by 0.6 ml of 0.6 M sucrose (F2). The supernatant from the 10,000*g* spin was overlaid onto F2, and the exact volume was recorded (x ml). The gradient was spun for 3 h at 180,000*g* at 4 °C. A blank gradient was made and spun at the same time as the sample containing the gradient. After the spin, the top of the gradient was removed and discarded (x ml – 0.6 ml = volume removed), leaving exactly 0.6 ml above F2. This was designated fraction 1 (F1). Fractions two and three were subsequently collected. Each fraction was diluted with ice-cold PBS and spun at 100,000*g* at 4 °C (Beckman Coulter, TLA 100.3) for 1 h to pellet the vesicles. Following spin completion, the supernatant was discarded, and pellets were collected in ice-cold PBS. The vesicles in fractions two to three was characterized by nanoparticle tracking analysis, immunoblotting, and TEM.

The EVs isolation from the cell culture media was completed as described by Palsa *et al.* (9) by sequential centrifugation. Briefly, the basal chamber media were collected and centrifuged at 1) 300*g* for 10 min to remove the cell pellets, and 2) the supernatant was centrifuged at 2000*g* for 10 min to remove the dead cell pellet. The resultant supernatant was centrifuged at 4000*g* for 30 min to remove the cell debris pellet. The resultant supernatant was collected in a 100K filter (Amicron Ultra-15, Centrifugal filters, Merck Millipore Ltd) to concentrate the media. The concentrated media were collected and centrifuged at 100,000*g* for 70 min (Beckman Coulter, TLA 100.3). The supernatant was discarded, and the pellet was washed with PBS and again centrifuged at 100,000*g* for 70 min, with the resultant EV pellet used for downstream processing.

### Transmission electron microscopy

TEM was performed as previously described (9). Briefly, 10 μl of EVs solution was placed on parafilm. Formvar-coated copper grids were then placed on top of the drops and incubated for 20 min. The copper grids were then incubated with a 4% solution of paraformaldehyde in 0.1 M PBS for 20 min, washed thrice with PBS for 1 min each, incubated with 1% glutaraldehyde in 0.1 M PBS for 5 min, washed with distilled water for 2 min, washed thrice with PBS for 2 min each, contrasted with 1% uranyl acetate for 20 s, and then observed by TEM (JEOL-1400).

### EVs subpopulations isolation

The EV subpopulation was performed as described previously (54). 400 μl of magnetic beads (Invitrogen, MSPB-6003) were washed three times using PBS, and then incubated with Biotin anti-mouse CD54 (1:50, 116103, Biolegend) or, as a control, Biotin Mouse IgG2b, κ Isotype Ctrl Antibody (1:50, 401204, Biolegend) on a shaker at room temperature for 1 h. The beads were then placed on the magnet for 1 min and washed with PBS. 600 μg of EVs (in 0.5 ml PBS) was used in the experiment, of which 300 μg was incubated with biotinylated mouse IgG2b, or ICAM-1 antibody-coupled magnetic beads at 4 °C overnight. After magnet absorption, the supernatant was transferred to a new tube for ultracentrifugation to obtain the EVs after depletion of ICAM^+^ EVs as the total EVs group. The magnetic beads bound with ICAM^+^EVs were collected and washed three times with PBS to obtain purified ICAM^+^ EVs. The same amounts of proteins (12 μg) for each group were loaded for Western blot analysis.

### Immunoblotting

Immunoblotting was performed as described previously (9). Briefly, isolated brain microvasculature was homogenized in NP-40 buffer that contained a protease inhibitor cocktail (Cell Signaling Technology, USA) and a phosphatase inhibitor cocktail (Sigma Aldrich, USA). After incubating on ice for 20 min, the homogenate was sonicated and centrifuged at 12,000 rpm for 10 min at 4 °C. The protein content of the supernatant was measured using the micro-BCA kit method. An equal amount of protein (30 μg) was fractionated on 4%–20% SDS-gels under reducing conditions and transblotted onto PVDF membranes. The blots were blocked with 5% fat-free dry milk and probed for CD63 (Thermo Fisher Scientific; 1:1000, 10628D), FPN1(Alpha diagnostic International, 1:1000, MTP-11S), ICAM-1(Invitrogen, 1:1000, MA5407) FTH1 (Cell Signaling Technology, 1:1000, 4393S), TfR1(Santa Cruz Biotechnology; 1:250, sc-65882), tsg 101 (Santa Cruz Biotechnology; 1:250, sc-7964), Calnexin (cell Signaling Technology; 1:1000, 2679), GFAP (Abcam, 1:1000, ab68428), Iba1 (Abacam, 1:1000, ab178846), IRP1 (Santa Cruz Biotechnology; 1:250, sc-166022) and IRP2 (Cell Signaling Technology, 37135; 1:1000), Vinculin (Sigma Millipore, 1:2000, V9131), and beta-actin (Sigma, 1:1000, A5441). A corresponding secondary antibody conjugated to HRP was employed (1:5000, GE Amersham), and bands were visualized using ECL reagents (PerkinElmer) on an Amersham Imager 600 (GE Amersham). The images were quantified with ImageJ software (NIH, Bethesda, MD, USA) and normalized to the respective loading controls, β-actin and vinculin. Proinflammatory cytokines IL-6 and TNF-alpha were measured using mouse IL-6 (Proteintech, KE10007) and mouse TNF-alpha (Proteintech, KE10002) enzyme-linked immunosorbent assay (ELISA) kits, in accordance with the manufacturer's protocol.

### Iron (^57^Fe) release studies

We have previously described the methodology for performing release assays (51). Briefly, huECs in serum-free media plated in a 12-well Transwell format were loaded with 100 μM ^57^Fe-ascorbic acid complex and incubated overnight at 37 °C. Following incubation, cells and chambers were rinsed three times with 1 × DPBS, and serum-free media was added, along with TNF-α (10 ng/ml) or IL-6 (50 ng/ml). These concentrations were selected based on previous studies. ^57^Fe release in EVs was measured after 24 h of incubation. As described before, tight junction formation and barrier permeability were measured *via* TEER and RITC signal.

### Inductively coupled plasma mass spectrometry (ICP-MS) for ^56^Fe and ^57^Fe analysis

ICP-MS is a highly sensitive instrument used to quantify the elements and isotopes of metals. We measured the concentration of ^56^Fe (the most common biological form of iron, brain tissue) and ^57^Fe (EVs). Samples were prepared for ICP-MS as described previously (40). Briefly, wet brains and EVs were digested overnight with ultra-pure metal-free nitric acid (2 ml) and 30% hydrogen peroxide (1 ml) at 60 °C. Samples were diluted in ultra-pure metal-free water. ^56^Fe and ^57^Fe concentrations were determined by ICP–MS against a standard (Millipore-Sigma # 68436). Results were expressed as μg/g of wet tissue weight.

### Nanoparticle tracking analysis

EVs quantification was performed using a NanoSight NS300 (Malvern Instruments Ltd, Malvern) previously (9). Briefly, EVs samples were diluted in 1 ml of particle-free water, and each sample was loaded by syringe pump into the NanoSight NS300 (Malvern Instruments Ltd, Malvern) set in scatter mode, and five 60-s videos were generated at 24.98 frames/sec. The size distribution and concentration of particles were calculated, and images were acquired using NanoSight software, version 3.2 (Malvern Instruments Ltd).

### Prussian Blue staining

Prussian Blue staining was performed on the non-denaturing gels to demonstrate that the H-ferritin from the brain EVs contained iron following a procedure described previously (55). Briefly, whole brain EVs were isolated and heated to 70 °C for 5 min and loaded onto the non-denaturing gels. Subsequently, non-denaturing gels were stained with a mixture (1:1v/v) of 2% potassium ferrocyanide (K4Fe (CN)6) and 2% 11.6N HCl prepared immediately before use and incubated overnight at room temperature, then rinsed with distilled water until reaching a close-to-neutral pH and clear bands appeared.

### Statistical analysis

All the data were presented as the mean ± SD. Statistical analysis was employed with the GraphPad Prism version 9.0 software. Comparison of differences between two or multiple groups by Student's unpaired *t* test and one-way ANOVA followed by Tukey’s *post hoc* analysis respectively. *p* < 0.05 was considered statistically significant.

## Data availability

All the data analyzed and generated during this study are included in this article. All raw data contained in the manuscript are available from the corresponding author upon request.

## Supporting information

This article contains supporting information (56, 57).

## Conflict of interest

The authors declare no conflict of interest.
